# The antibody response in the bovine mammary gland is influenced by the adjuvant and the site of subcutaneous vaccination

**DOI:** 10.1186/s13567-018-0521-2

**Published:** 2018-03-01

**Authors:** Eveline M. Boerhout, Ad P. Koets, Tanja G. T. Mols-Vorstermans, Piet J. M. Nuijten, Mathieu J. H. Hoeijmakers, Victor P. M. G. Rutten, Jetta J. E. Bijlsma

**Affiliations:** 1 Ruminants Research and Development, MSD Animal Health, Wim de Körverstraat 35, 5830 AA Boxmeer, The Netherlands; 2 Department of Bacteriology and Epidemiology, Central Veterinary Institute Part of Wageningen UR, Edelhertweg 15, PO box 65, 8200 AB Lelystad, The Netherlands; 3 Department of Farm Animal Health, Faculty of Veterinary Medicine, Utrecht University, Yalelaan 7, 3584 CL Utrecht, The Netherlands; 4 Global Clinical Research, MSD Animal Health, Wim de Körverstraat 35, 5830 AA Boxmeer, The Netherlands; 5 Department of Infectious Diseases and Immunology, Faculty of Veterinary Medicine, Utrecht University, Yalelaan 1, 3584 CL Utrecht, The Netherlands; 6 Department of Veterinary Tropical Diseases, Faculty of Veterinary Science, University of Pretoria, Private Bag X04, Onderstepoort, 0110 South Africa; 7 Discovery and Technology, MSD Animal Health, Wim de Körverstraat 35, 5830 AA Boxmeer, The Netherlands

## Abstract

Intramammary infections in cattle resulting in mastitis have detrimental effects on cows’ well-being, lifespan and milk production. In the host defense against *S. aureus* mastitis antibodies are thought to play an important role. To explore potential ways to increase antibody titers in the bovine mammary gland the effects of various adjuvants on the magnitude, isotype, and neutralizing capacity of antibodies produced following subcutaneous vaccine administration at different immunization sites were analyzed. In this study, α-toxoid was used as a model antigen and formulated in three different alum-based adjuvants: Alum–Saponin, Alum–Oil, and Alum–Saponin–Oil. Vaccines were administered near the suspensory ligament of the udder or in the lateral triangular area of the neck. At both immunization sites, immunization with α-toxoid in Alum–Saponin–Oil resulted in higher specific antibody titers in milk and serum as compared with Alum–Oil and Alum–Saponin, without favoring an IgG1, IgG2, or IgA response. Furthermore, the neutralizing capacity of milk serum and serum following immunization near the udder and in the neck was higher when Alum–Saponin–Oil was used as adjuvant compared with Alum–Oil and Alum–Saponin. Prime immunizations near the udder effectively increased both antibody isotype titers and neutralization titers, while prime plus boost immunizations were required to induce similar effects following immunization in the neck. Results indicate that subcutaneous administration of an Alum–Saponin–Oil based vaccine near the udder could be further explored for the development of a one-shot vaccination strategy to efficiently increase intramammary antibody responses.

**Electronic supplementary material:**

The online version of this article (10.1186/s13567-018-0521-2) contains supplementary material, which is available to authorized users.

## Introduction

Intramammary infections (IMI) in cattle often result in mastitis with detrimental effects on the cows’ well-being, lifespan and milk production [1]. Vaccination represents one of the most studied and sought after tools to prevent bovine mastitis [2–4]. However, despite numerous attempts to develop an effective vaccine, commercially available vaccines are scarce and their evaluation under field conditions showed limited protection only [5, 6]. *Staphylococcus aureus* (*S. aureus*) represents one of the major mastitis causing pathogens. Although immune correlates of protection against *S. aureus* mastitis have not yet been well established, high *S. aureus*-specific IgG1 levels in milk have been associated with reduced growth of *S. aureus* both in vivo and in vitro, suggesting a role for this antibody isotype in the hosts defense against *S. aureus* [7–9]. IgG1 is the most abundantly present antibody isotype in the healthy mammary gland and it facilitates phagocytosis by macrophages, the predominant leukocytes in normal milk [10, 11]. IgG2, which is accumulated in inflamed tissue, promotes phagocytosis by neutrophils and is thought to play a role during later stages of IMI [12, 13]. IgA can be found in mucosal secretions such as milk and prevents mucosal infections by agglutinating microbes [14, 15]. Several studies suggest that antibodies also play a role in the host defense by neutralizing immune evasion proteins which are secreted by *S. aureus* during IMI [16, 17]. The failure of presently available vaccines to protect cattle against IMI might be due to an insufficient capacity to induce strong, neutralizing antibody responses in the mammary gland. Both the magnitude and neutralizing capacity of antibodies are influenced by the route and site of vaccine administration [18–20]. In cattle, subcutaneous (SC) immunization near the supramammary lymph node positively influences the antibody response in both milk and serum [18, 21]. The aim of this study was to determine whether the site of the subcutaneous immunization in combination with the use of various adjuvants influences the magnitude, isotype, and neutralizing capacity of antibodies in milk. Therefore, cows were immunized near the suspensory ligament of the udder (in this study referred to as the udder) or in the lateral triangular area of the neck (in this study referred to as the neck). The α-toxoid of *S. aureus*, known to induce functional antibody responses [22–25], was used as model antigen. Since the ability of adjuvants to modulate antibody responses is generally appreciated and widely exploited in different immunization strategies [26–28], α-toxoid was formulated in alum-based adjuvants supplemented with either saponin, oil, or both in order to determine the influence of the adjuvant on the antibody response induced.

## Materials and methods

### Animals

A total of 48 clinically healthy first-lactation Holstein-Frisian cows between 2 and 3 years of age and 5–6 months in lactation were used in this study. The geomean milk production of the enrolled cows was 24.2 L/day (range 18.6–32.6 L/day). All cows had a quarter milk SCC below 100 000 cells/mL upon enrollment and no history of mastitis. Cows were randomly divided over 6 groups of 8 cows. Animals were housed in a free stall barn and fed according to their requirements [29] (For Farmers Hendrix, Lochem, The Netherlands). Water was supplied ad libitum. Cows were milked twice a day and the milk yield (kg/day) was recorded throughout the study. Post-milking teat disinfection was practiced with a 0.5% iodine disinfectant. Rectal temperatures were measured pre-immunization, 4 h and 1, 2, and 3 days post-prime and boost-immunization. Cows were daily monitored for signs of clinical mastitis.

### Experimental vaccine and immunization procedure

To produce α-toxoid the *S. aureus* DU1090 strain, which contains the pDU1212[H35R] plasmid encoding a genetically detoxified α-toxin [30], was cultured in Todd Hewitt Broth medium (MSD-AH, Boxmeer, The Netherlands) for 24 h at 37 °C. Culture medium was filtered (0.2 µm) to remove cells and α-toxoid was concentrated over a 10 kDa ultrafilter. Purity and concentration were checked by SDS-PAGE and Coomassie blue staining. The experimental vaccine consisted of 15 µg α-toxoid per dose (4 mL) formulated in a proprietary alum-based adjuvant (Brenntag, Dordrecht, The Netherlands) supplemented with either saponin (QuilA; Brenntag), light mineral oil, or both (MSD-AH). Each vaccine was administered subcutaneously to two groups: one group received 2 mL of the vaccine on each the left and right side of the suspensory ligament of the udder at both prime and boost immunizations, while the other group received 4 mL of the vaccine in the lateral triangular area on the left side of the neck for the prime immunization, and 4 mL on the right side of the neck for the boost immunization. Prime and boost immunizations were administered 6 weeks apart. Vaccines were administered using 18G needles (BD Microlance™, Broek op Langedijk, The Netherlands).

### Local reaction scores

Local reactions to the experimental vaccines were scored pre-immunization, and 1, 2, 3, 7, and 14 days post-prime and boost-immunization, based on swelling size estimations at the injection site and classified in four categories: swelling with a diameter of (1) 0–5 cm, (2) 5–10 cm, (3) 10–15 cm, or (4) > 15 cm. Since immunizations near the udder were administered on both the left and right side of the udder each time for prime and boost immunizations, the sum of the swelling size on both immunization sites was used as final score.

### Milk and blood sampling

Milk and blood samples were collected pre-immunization, 4 weeks post-prime and 2 weeks post-boost immunization [25]. For each individual cow, a representative aliquot (10 mL) of the mixed morning milk yield of all four quarters was collected and transported to the laboratory at ambient temperature. Part of the milk sample was centrifuged for 15 min at 2000 × *g* to collect milk serum as whole milk interfered with the α-toxin neutralization assay. Blood was collected from the coccygeal vein using a sterile blood collection system (BD Vacutainer, Beckton Dickinson B.V., Breda, The Netherlands). Following coagulation, blood was centrifuged for 10 min at 3000 ×* g* to collect blood serum (in this study referred to as serum). Milk, milk serum, and serum samples were stored at −20 °C until further analysis.

### Titers and isotypes of α-toxin specific antibodies measured by ELISA

α-Toxin specific antibody isotype titers were determined by ELISA. NUNC MaxiSorp plates (eBioscience, Hatfield, UK) were coated overnight at 4 °C with α-toxin (1 µg/mL for IgG1 and IgG2, and 0.5 µg/mL for IgA; Sigma-Aldrich, St. Louis, MO, USA). Samples were added in threefold serial dilutions and incubated for 1 h at 37 °C. Eight replicates of an in-house negative control serum were taken along. Horseradish-peroxidase-conjugated sheep-anti-bovine IgG1, IgG2, and IgA conjugates (Catalogue numbers A10-116p, A10-117P, and A10-121P; Bethyl Laboratories Inc., Montgomery, Texas, USA) were used in 1:500, 1:2000, and 1:1000 dilutions, respectively. Tetramethylbenzidine was used as a substrate and reactions were stopped by adding sulfuric acid. Extinctions (450 nm) were measured on a Tecan SUNRISE device using XFluor4 Software Version V4.51-I4 (Tecan Group Ltd., Männedorf, Switzerland). Antibody titers were determined using CaSpEx Software Version 1.12 (MSD, Proprietary Software) and defined as the Log_2_ dilution of the sample that would give the same absorbance as 1.6 (IgG1 and IgG2) or 1.75 (IgA) times the average OD of the negative controls.

### α-Toxin neutralization assay

Samples were analyzed for their capacity to neutralize α-toxin, thereby preventing α-toxin mediated erythrocyte lysis, in twofold serial dilutions. Rabbit blood was collected and immediately mixed 1:1 with Alsever’s solution (MSD-AH) to prevent clotting. Erythrocytes were harvested by centrifugation for 10 min at 2000 × *g*. Erythrocytes were washed twice with 0.04 M PBS (MSD-AH), and dissolved in a volume of PBS equal to the volume of the original blood sample. Samples were incubated with 50 µL α-toxin (1.6 µg/mL; Sigma-Aldrich) for 30 min at 37 °C while gently shaking. Then, erythrocytes (50 µL) were added and samples were incubated for 1 h at 37 °C. For maximal and minimal lysis, erythrocytes were incubated with and without α-toxin, respectively, in the absence of milk serum and serum. Following incubation, supernatant was transferred to a clean microtiter plate. Extinctions (OD 545 nm) were measured on a Tecan SUNRISE device. Neutralization titers were determined using CaSpEx and defined as the Log_2_ sample dilution that resulted in 25% lysis inhibition based on the average OD of the samples with maximal and minimal lysis.

### Statistical analyses

Statistical analyses were performed using the statistical software package SAS Version 9.3 (SAS Institute Inc., Cary, NC, USA). An ANOVA linear mixed model was used to identify effects of the different adjuvants and/or immunization sites on titer increases post-immunization. A full interaction model including “adjuvant” (Alum–Saponin/Alum–Oil/Alum–Saponin–Oil), “site” (udder/neck), and “time” (pre-immunization/post-prime/post-boost) was used. Pre-immunization titers were included as covariates and “cow” was added as a random effect. The likelihood ratio test was used to select the most parsimonious variance–covariance structure. An unstructured covariance structure was used for milk IgA antibody titers and milk neutralization titers. A compound symmetry structure was used for all other analyses. Tests were two-sided using a significance level of 0.05. Correlations between titers were estimated by the Spearman’s correlation coefficient. For graphical presentation of the data GraphPad Prism Software Version 7 (GraphPad Software Inc., La Jolla CA, USA) was used.

## Results

### Local reaction scores post-immunization

Immunizations had no effect on the body temperature and daily milk yield (data not shown). Local reaction scores of 4 and 3 were observed post-immunization with Alum–Saponin near the udder and in the neck, respectively. Scores decreased from 1 week post-immunization onwards. Local reaction scores post-immunization with Alum–Oil and Alum–Saponin–Oil resulted in scores of 4 near the udder and in the neck. Scores persisted throughout the experimental period (Additional file 1).

### α-Toxin specific antibody isotype titers

Significant mean differences in milk and serum IgG1, IgG2, and IgA antibody titers (AT) and neutralization titers (NT) post-immunization with different adjuvants and/or via different immunization sites are shown in Additional file 3. Only significant differences in mean titers of at least 1.5 Log_2_ (Table 1) were considered biologically relevant and are addressed below.

**Table 1:** **Differences in least square means (Log**_**2**_ **) of α-toxin specific antibody isotype titers and neutralization titers in milk and serum**

Effect	Adjuvant	Site	Time	# Adjuvant^a^	# Site^a^	# Time^a^	Estimate	Lower^b^	Upper^b^	*p* value	Sample	Titer
Adjuvant	Alum–Saponin–Oil	–	–	Alum–Saponin	–	–	+1.6	+0.7	+2.5	0.0012	Milk	IgG2
				Alum–Oil	–	–	+1.5	+0.9	+2.1	< 0.0001	Serum	IgG2
Adjuvant × time	Alum–Saponin–Oil	–	Prime	Alum–Saponin–Oil	–	Boost	−2.3	−3.1	−1.5	< 0.0001	Milk	IgG1
							−2.0	−2.3	−1.7	< 0.0001	Serum	IgA
							−1.8	−2.3	−1.3	< 0.0001	Serum	IgG1
		–	Boost	Alum–Saponin	–	Boost	+2.6	+1.9	+3.4	< 0.0001	Serum	IgG1
							+2.5	+1.4	+3.5	< 0.0001	Milk	IgG1
							+1.9	+1.3	+2.6	< 0.0001	Serum	IgA
							+1.6	+0.7	+2.5	0.0011	Milk	IgA
		–	Boost	Alum–Oil	–	Boost	+1.8	+1.0	+2.6	< 0.0001	Serum	IgG1
Adjuvant × site	Alum–Saponin–Oil	Udder	–	Alum–Saponin	Udder	–	+2.6	+1.4	+3.8	< 0.0001	Milk	IgG1
		Udder	–	Alum–Oil	Udder	–	+1.9	+0.6	+3.3	0.0069	Milk	IgG1
	Alum–Oil	Neck	–	Alum–Saponin	Neck	–	+2.1	+0.9	+3.2	0.0009	Milk	IgG1
		Udder	–	Alum–Oil	Neck	–	−2.1	−3.4	−0.9	0.0014	Milk	IgG1
Site × time	–	Neck	Prime	–	Neck	Boost	−2.0	−2.4	−1.6	< 0.0001	Milk	IgG2
							−1.9	−2.6	−1.3	< 0.0001	Milk	IgG1
							−1.8	−2.2	−1.4	< 0.0001	Milk	NT

This table lists only statistically significant comparisons with an effect size of at least 1.5 Log_2_.

NT: neutralization titer.

^a^# compared to.

^b^Confidence interval (95%).

**Figure 1:**
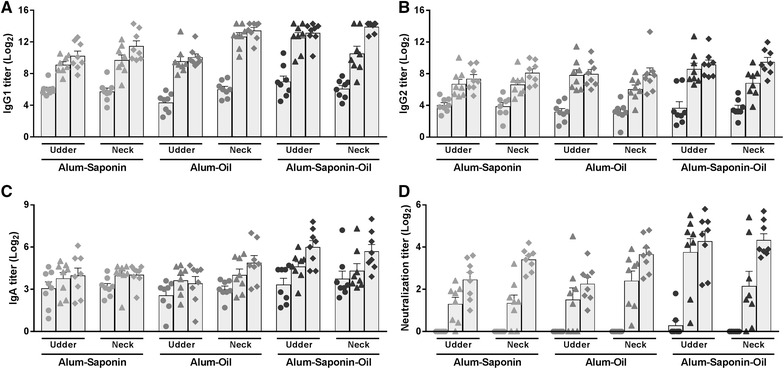
**α-Toxin specific antibody isotype titers and neutralization titers in milk.** Specific antibody isotype titers were measured in an α-toxin specific ELISA. The neutralization capacity of milk serum was analyzed in an α-toxin neutralization assay. Milk **A** IgG1, **B** IgG2, and **C** IgA antibody titers and **D** milk serum neutralization titers pre-immunization (filled circle), post-prime (filled triangle), and post-boost (filled diamond) immunization are expressed as the mean ± SEM per group. Significant mean titer differences are given in Table 1 and Additional file 3.

Pre- and post-immunization milk IgG1, IgG2, and IgA AT are depicted in Figures 1A–C, respectively. Adjuvant effects on the titer increase following prime plus boost immunization were observed (adjuvant effect). The use of Alum–Saponin–Oil resulted in higher mean IgG2 AT with differences of 1.6 Log_2_ for milk (Figure 1B) and 1.5 Log_2_ for serum (Additional file 2B), as compared with Alum–Saponin and Alum–Oil, respectively.

Time-dependent adjuvant effects were also observed (adjuvant * time effect). Boost immunizations with Alum–Saponin–Oil resulted in 2.3 and 1.8 Log_2_ higher mean IgG1 AT in milk (Figure 1A) and serum (Additional file 2A), respectively, and 2.0 Log_2_ higher mean IgA AT in serum (Additional file 2C), as compared with titers induced by prime immunizations. When using Alum–Saponin–Oil as adjuvant, boost immunizations resulted in 2.6 and 1.8 Log_2_ higher mean IgG1 AT in serum as compared with boost immunizations with Alum–Saponin and Alum–Oil, respectively (Additional file 2A). Furthermore, mean IgG1 AT in milk (Figure 1A) and IgA AT in both milk (Figure 1C) and serum (Additional file 2C) were 2.5, 1.6, and 1.9 Log_2_ higher post-boost immunization with Alum–Saponin–Oil as compared with Alum–Saponin, respectively.

Some adjuvant effects were immunization site dependent (adjuvant * site effect). When administered near the udder, mean IgG1 AT in milk (Figure 1A) were 2.6 and 1.9 Log_2_ higher post-immunization with Alum–Saponin–Oil as compared with Alum–Saponin and Alum–Oil, respectively. When administered in the neck, mean IgG1 AT in milk were 2.1 Log_2_ higher post-immunization with Alum–Oil as compared with Alum–Saponin. Using Alum–Oil as adjuvant, vaccine administration in the neck resulted in 2.1 Log_2_ higher mean IgG1 AT in milk compared with administration near the udder. Boost immunizations showed significant effects on milk IgG1 and IgG2 AT (Figures 1A and B) when administered in the neck with estimated mean titer increases of 1.9 and 2.0 Log_2_, respectively (site * time effect). Although not significant for all parameters, prime immunizations with Alum–Saponin–Oil near the udder increased milk IgG1 (1.59 Log_2_; *p* = 0.0305), IgG2 (1.70 Log_2_; *p* = 0.0168) and IgA (0.53 Log_2_; *p* = 0.2718) AT and NT (1.43 Log_2_; *p* = 0.0651) with greater magnitude as compared with prime-immunization in the neck. Similar results were obtained for serum IgG1 (1.26 Log_2_; p = 0.0345), IgG2 (1.15 Log_2_; *p* = 0.0163) and IgA (0.98 Log_2_; *p* = 0.0418) AT and NT (1.52 Log_2_; *p* = 0.0003) (Additional files 2A–C).

### α-Toxin neutralization capacity of milk serum and blood serum

The neutralization titers (NT) of pre- and post-immunization milk serum are depicted in Figure 1D. No adjuvant dependent effect on the NT was observed. The site of vaccine administration influenced the milk serum NT in a time dependent manner (site * time effect). Boost immunizations resulted in higher milk serum NT when administered in the neck as compared with administration near the udder with an estimated mean titer difference of 1.8 Log_2_. Comparable results were observed for the neutralization capacity of serum (Table 1; Additional file 2D, Additional file 3).

### Titer correlations

**Figure 2:**
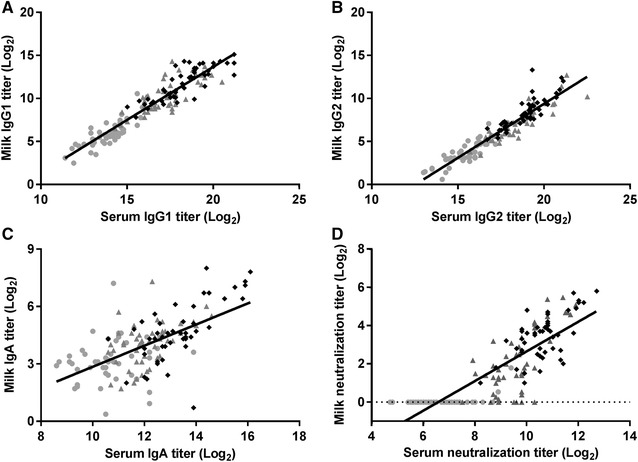
**Correlation between milk and serum titers.** Plots represent correlations between α-toxin specific titers in milk and serum for **A** IgG1, **B** IgG2, **C** IgA, and **D** neutralization titers as measured pre-immunization (filled circle), post-prime (filled triangle) and post-boost (filled diamond) immunization. Spearman correlation coefficients were **A** 0.928, **B** 0.954, **C** 0.446, and **D** 0.876.

The comparable dynamics of AT and NT in milk and serum post-immunization are reflected in the high correlation between milk and serum titers (Figure 2). IgG1 and IgG2 milk and serum AT showed strong Spearman correlation coefficients of 0.928 and 0.954, respectively. NT of milk serum and serum were also strongly correlated with a Spearman correlation coefficient of 0.876. IgA AT in serum and milk were not correlated and showed a Spearman correlation coefficient of only 0.446.

**Figure 3:**
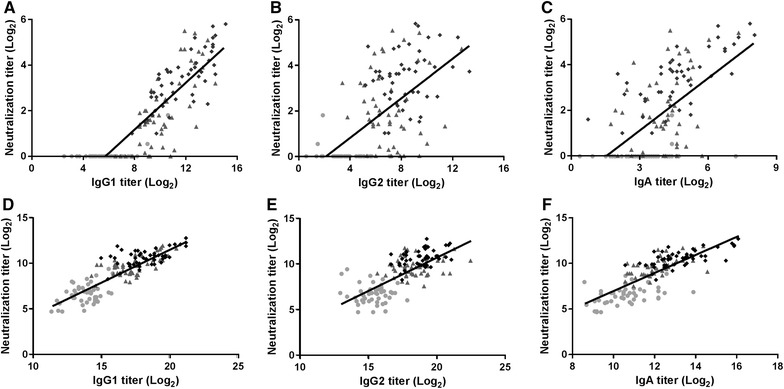
**Correlation between α-toxin specific antibody isotype titers and neutralization titers.** Plots represent the correlation between milk **A** IgG1, **B** IgG2, and **C** IgA titers with their respective milk neutralization titers, and the correlation between serum **D** IgG1, **E** IgG2, and **F** IgA titers with their respective serum neutralization titers. Titers pre-immunization (filled circle), post-prime (filled triangle) and post-boost (filled diamond) immunization are depicted. Spearman correlation coefficients were **A** 0.838, **B** 0.727, **C** 0.583, **D** 0.893, **E** 0.659, and **F** 0.621.

Correlations between the IgG1, IgG2 and/or IgA AT and their corresponding NT pre- and post-immunization were also analyzed. The highest correlation with NT was observed for IgG1 milk and serum titers with Spearman correlation coefficients of 0.838 and 0.893, respectively (Figures 3A and D). Spearman correlation coefficients for α-toxin-specific IgG2 titers in milk and serum and their respective NT were 0.727 and 0.659 (Figures 3B and E). The IgG1:IgG2 ratios were not influenced by the type of adjuvant and/or site of SC vaccine administration and remained unchanged in the course of the experiment. Spearman correlation coefficients for α-toxin specific IgA AT in milk and serum and their respective NT were 0.583 and 0.621 (Figures 3C and F).

## Discussion

Alum-based adjuvants are widely used in human and veterinary vaccines and known to induce effective, primarily antibody-mediated, immune responses [31–33]. In the present study, alum-based adjuvants were supplemented with saponin, a well-recognized potent stimulator of both humoral and cellular immune responses [34–36], or/and an oil emulsion, which is thought to provide a depot effect at the injection site resulting in sustained antigen release providing long term immune stimulation [37]. The synergistic effects of alum and saponin have previously been reported in sheep where immunization with a Bacteroides antigen formulated in Alum–Saponin induced higher AT and NT as compared with formulation in alum only [38]. Supplementation of an alum-based adjuvant with oil increased the humoral and cellular immune responses in mice with greater magnitude and better neutralizing capacity as compared with an adjuvant based on alum only [39]. Saponin and oil can also act synergistically as shown in a murine model where increased AT were observed following supplementation of the oil adjuvanted commercial FMD vaccine and an experimental oil-based HIV-1 vaccine with saponin [35, 40]. Results of the present study show that supplementation of an alum-based adjuvant with both saponin and oil resulted in higher AT compared with immunization with Alum–Oil and Alum–Saponin, respectively.

In the present study, saponin did not specifically favor the induction of IgG2 as shown by unchanged IgG1:IgG2 ratios in the course of the experiment. Since pre-immunization titers indicate previous contact with *S. aureus*, saponin may have been ineffective in redirecting the already established immune response. Alternatively, the stimulating effects of alum on the humoral response might have prevented saponin to direct the immune system to a more Th1 cellular based response.

Previous studies in a murine model show that the type of immune response induced is influenced by the site of SC antigen administration [20, 41, 42]. Previous studies in cattle describe no differences in specific AT in both milk and serum following vaccine administration near the udder or in the neck using a polymer-based adjuvant or an oil-in-water adjuvant [21, 43]. In the present study, the site of vaccine administration did not influence the IgG1:IgG2 ratios in the course of the experiment. Although the use of Alum–Oil resulted in increased IgG1 AT to a greater magnitude post-immunization in the neck compared with immunization near the udder, this effect was not reflected in the IgG2 and IgA AT and NT. For Alum–Saponin and Alum–Saponin–Oil, no immunization site specific effects on overall AT and NT were observed.

Independent of the adjuvant used, the effect of boost immunizations was strongest following administration in the neck. As shown in a murine model, the major reservoir of memory B-cells is the spleen, but subsets of memory B-cells are retained in draining lymphoid sites [44, 45]. Following secondary antigen encounter, memory B-cells are rapidly reactivated and stimulated to proliferate and differentiate into antibody secreting plasma cells. Since *S. aureus* is frequently isolated from external orifices and the teat skin [46, 47], the supramammary lymph nodes are likely to have acted as draining LN following natural contact with *S. aureus* resulting in the priming of resident immune cells. In contrast, since skin infections caused by *Staphylococcus* ssp. in the neck area, drained by the prescapular LNs, are highly uncommon [48], it is unlikely that the prescapular LNs have previously encountered *S. aureus* antigens. Therefore, prime immunizations in the neck may have resulted in the generation and local presence of memory B-cells able to mount a recall response post-boost immunization to a greater extend compared to prime immunization near the udder which apparently already acted as a booster type of immunization. Interestingly, a prime immunization near the udder with Alum–Saponin–Oil was sufficient to increase IgG1 and IgG2 AT and NT with similar magnitude compared with prime plus boost immunizations in the neck, suggesting highly effective re-activation of memory B-cells by this adjuvant. This effect was not observed for IgA which in this case did respond in a prime boost fashion. This can indicate a relative lower number of these type of memory B-cells at this site which may be relevant given the role of IgA on mucosal surfaces. Alternatively, the magnitude of the immune response might have been influenced by the antigen dose and number of LNs simultaneously targeted as a consequence of our immunization strategy. Since the vaccine was divided over the left and right site of the udder for both prime and boost immunizations, a low antigen concentration per injection was administered, targeting both SMLNs. In contrast, immunizations in the neck resulted in the presentation of a higher antigen dose to the left (prime) or right (boost) prescapular LN. Although antigen presentation might not be exclusively restricted to the draining LN on the ipsilateral site, the induction of an immune response in the contralateral LN due to antigen relocation or migration of antigen presenting cells cannot be excluded [49]. Additional studies with similar antigen doses and number of injections are required to determine their possible role in the AT and NT dynamics observed in this study.

Regardless of the adjuvant and immunization site, a high correlation between milk and serum α-toxin specific AT were observed suggesting antibody exchange between the systemic circulation and the mammary gland. Pre-immunization milk AT did not correlate with NT in milk serum, suggesting either the absence of neutralizing antibodies or NT below the detection limit in the neutralization assay. Alternatively, immunizations might have induced antibodies with higher neutralizing capacities compared with antibodies induced following natural contact with *S. aureus* [16]. In pre-immunization serum α-toxin specific AT as well as NT were observed. Correlation coefficients between milk and serum AT and NT were higher for IgG1 as compared with IgG2 and IgA, which might suggest that neutralization of α-toxin is predominantly mediated by IgG1. Further tests with purified antibody isotypes are needed to provide additional proof.

In conclusion, in the attempts to develop an efficacious vaccine against bovine *S. aureus* mastitis, Alum–Saponin–Oil should be considered as adjuvant since it efficiently stimulates the induction of AT, favoring IgG1, IgG2, and IgA responses, and NT. While prime immunizations with Alum–Saponin–Oil near the udder resulted in high titer increases, immunization in the neck required a prime-boost regimen to induce similar titers. This implies that, when subcutaneously administered near the udder, a one-shot vaccination strategy with Alum–Saponin–Oil may be sufficient to efficiently increase intramammary antibody responses.

## Additional files

**Additional file 1.** **Local reaction scores post-immunization.** Local reaction scores (A) near the udder and (B) in the neck following prime (day 0) and boost (day 42) immunizations are depicted. Fig A shows the sum of the local reaction scores observed at the immunization site on the left and right side of the udder. Fig B shows the local reaction scores observed on the left side of the neck post-prime immunization and on the right side of the neck post-boost immunization. Data is expressed as the mean ± range per group for Alum–Saponin (filled circle), Alum–Oil (filled triangle), and Alum–Saponin–Oil (filled diamond).

**Additional file 2.** **α-Toxin specific antibody isotype titers and neutralization titers in serum.** Specific antibody isotype titers were measured in an α-toxin specific ELISA. The neutralization capacity of serum was analyzed in an α-toxin neutralization assay. Serum (A) IgG1 and (B) IgG2 antibody titers and (C) serum neutralization titers pre-immunization (filled circle), post-prime (filled triangle), and post-boost (filled diamond) immunization are expressed as the mean ± SEM per group. Significant mean titer differences are given in Table 1 and Additional file 3.

**Additional file 3.** **Differences in least square means (Log**_**2**_ **) of α-toxin specific antibody isotype titers and neutralization titers.**
