# Supplementary figures and images for: The antibody response in the bovine mammary gland is influenced by the adjuvant and the site of subcutaneous vaccination
Source: Vet Res. 2018 Mar 1;49:25. doi: 10.1186/s13567-018-0521-2 (PMC5831572; doi:10.1186/s13567-018-0521-2)

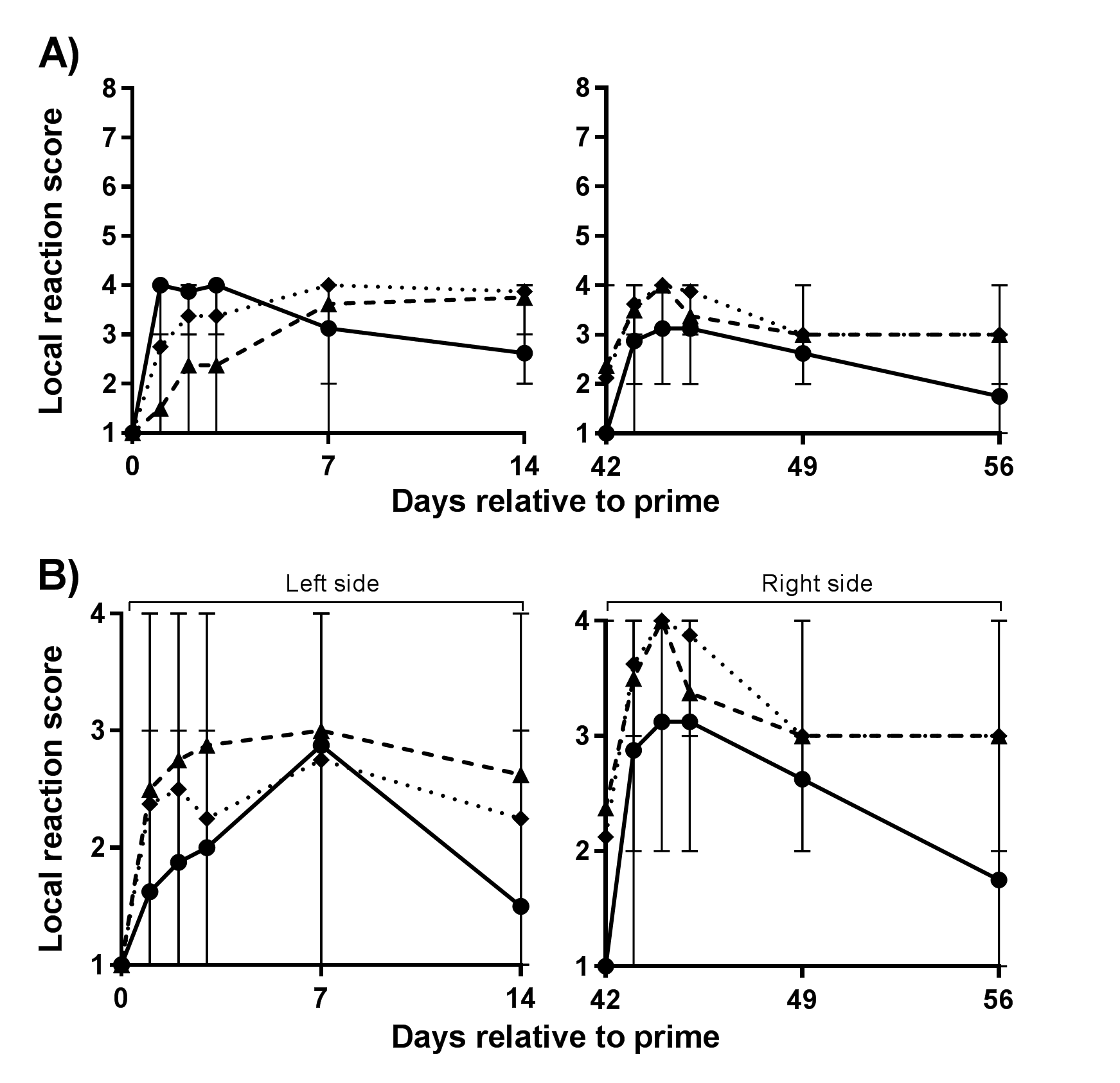

Supplement: Supplementary file 1 — Additional file 1. Local reaction scores post-immunization. Local reaction scores (A) near the udder and (B) in the neck following prime (day 0) and boost (day 42) immunizations are depicted. Fig A shows the sum of the local reaction scores observed at the immunization site on the left and right side of the udder. Fig B shows the local reaction scores observed on the left side of the neck post-prime immunization and on the right side of the neck post-boost immunization. Data is expressed as the mean ± range per group for Alum–Saponin (filled circle), Alum–Oil (filled triangle), and Alum–Saponin–Oil (filled diamond). [file 13567_2018_521_MOESM1_ESM.tif]

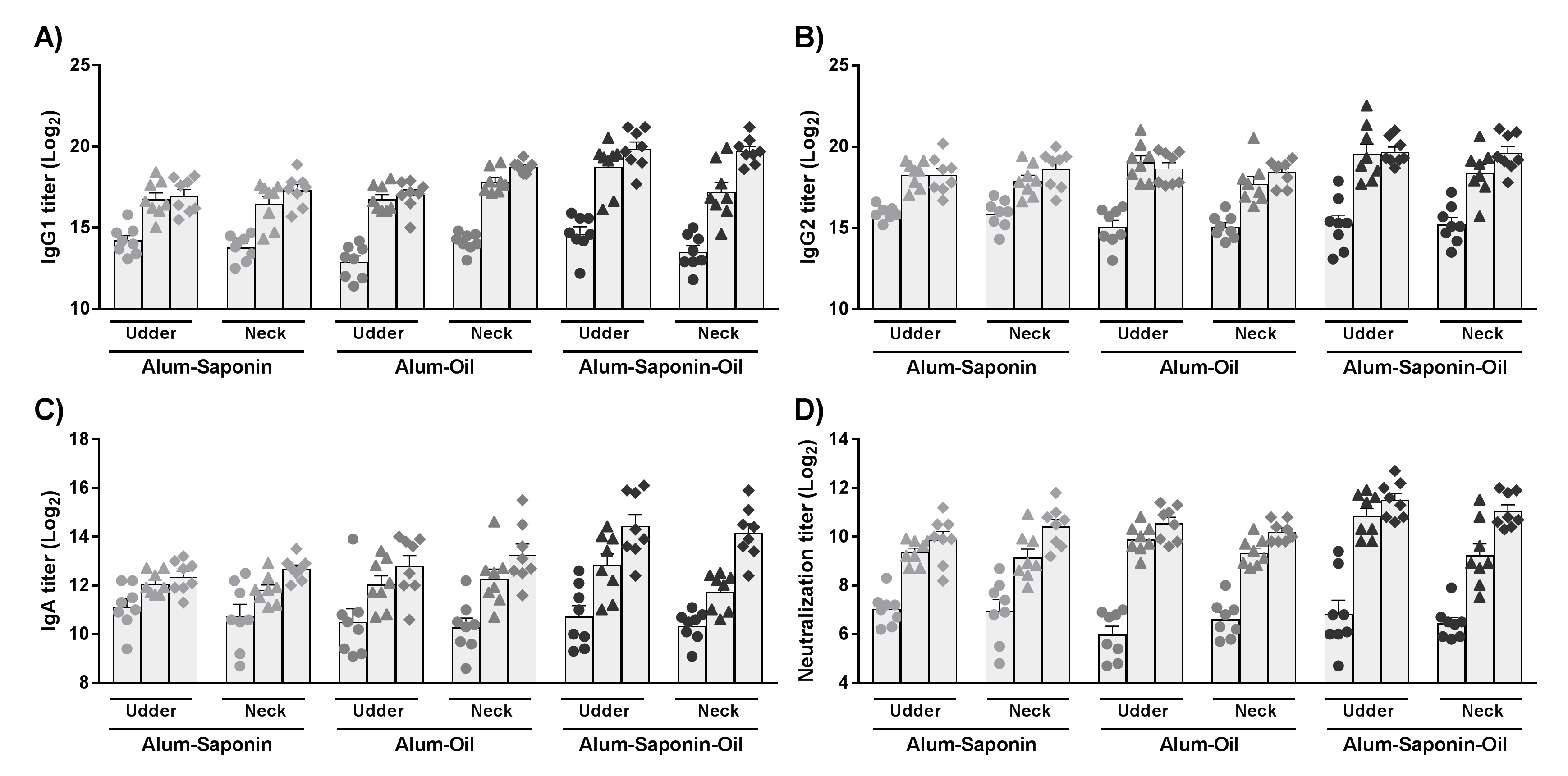

Supplement: Supplementary file 2 — Additional file 2. α-Toxin specific antibody isotype titers and neutralization titers in serum. Specific antibody isotype titers were measured in an α-toxin specific ELISA. The neutralization capacity of serum was analyzed in an α-toxin neutralization assay. Serum (A) IgG1 and (B) IgG2 antibody titers and (C) serum neutralization titers pre-immunization (filled circle), post-prime (filled triangle), and post-boost (filled diamond) immunization are expressed as the mean ± SEM per group. Significant mean titer differences are given in Table 1 and Additional file 3. [file 13567_2018_521_MOESM2_ESM.tif]
