# Supplementary material for: The antibody response in the bovine mammary gland is influenced by the adjuvant and the site of subcutaneous vaccination
Source: Vet Res. 2018 Mar 1;49:25. doi: 10.1186/s13567-018-0521-2 (PMC5831572; doi:10.1186/s13567-018-0521-2)
Supplement: Supplementary file 3 — Additional file 3. Differences in least square means (Log 2 ) of α-toxin specific antibody isotype titers and neutralization titers. [file 13567_2018_521_MOESM3_ESM.docx]

**Additional file 3 Differences of least square means of α-toxin specific antibody isotype titers and neutralization titers in serum and milk.**

| Effect | Adjuvant | Site | Time | # Adjuvant^a^ | # Site^a^ | # Time^a^ | Estimate | Lower^b^ | Upper^b^ | *p*-value | Sample | Titer |
| --- | --- | --- | --- | --- | --- | --- | --- | --- | --- | --- | --- | --- |
| Adjuvant | Alum-Saponin-Oil | - | - | Alum-Saponin | - | - | +1.6 | +0.7 | +2.5 | 0.0012 | Milk | IgG2 |
|  |  |  |  |  |  |  | +1.4 | +0.6 | +2.2 | 0.0012 | Milk | NT |
|  |  |  |  |  |  |  | +1.1 | +0.6 | +1.5 | <0.0001 | Serum | NT |
|  |  |  |  |  |  |  | +0.8 | +0.2 | +1.3 | 0.0102 | Serum | IgG2 |
|  | Alum-Saponin-Oil | - | - | Alum-Oil | - | - | +1.5 | +0.9 | +2.1 | <0.0001 | Serum | IgG2 |
|  |  |  |  |  |  |  | +1.1 | +0.3 | +1.9 | 0.0109 | Milk | NT |
|  |  |  |  |  |  |  | +0.6 | +0.1 | +1.1 | 0.0198 | Serum | NT |
|  | Alum-Oil | - | - | Alum-Saponin | - | - | +0.7 | +0.2 | +1.3 | 0.0156 | Serum | IgG2 |
| Adjuvant * Time | Alum-Saponin-Oil | - | Prime | Alum-Saponin-Oil | - | Boost | -2.3 | -3.1 | -1.5 | <0.0001 | Milk | IgG1 |
|  |  |  |  |  |  |  | -2.0 | -2.3 | -1.7 | <0.0001 | Serum | IgA |
|  |  |  |  |  |  |  | -1.8 | -2.3 | -1.3 | <0.0001 | Serum | IgG1 |
|  |  |  |  |  |  |  | -1.4 | -1.8 | -0.9 | <0.0001 | Milk | IgA |
|  | Alum-Oil | - | Prime | Alum-Oil | - | Boost | -0.9 | -1.7 | -0.1 | 0.0298 | Milk | IgG1 |
|  |  |  |  |  |  |  | -0.9 | -1.2 | -0.6 | <0.0001 | Serum | IgA |
|  |  |  |  |  |  |  | -0.6 | -1.1 | -0.1 | 0.0231 | Serum | IgG1 |
|  | Alum-Saponin | - | Prime | Alum-Saponin | - | Boost | -1.1 | -1.9 | -0.3 | 0.0076 | Milk | IgG1 |
|  |  |  |  |  |  |  | -0.6 | -0.9 | -0.2 | 0.0014 | Serum | IgA |
|  |  |  |  |  |  |  | -0.5 | -1.0 | -0.0 | 0.0349 | Serum | IgG1 |
|  | Alum-Saponin-Oil | - | Prime | Alum-Saponin | - | Prime | +1.4 | +0.6 | +2.1 | 0.0012 | Serum | IgG1 |
|  |  |  |  |  |  |  | +1.3 | +0.3 | +2.3 | 0.0125 | Milk | IgG1 |
|  | Alum-Oil | - | Prime | Alum-Saponin | - | Prime | +1.4 | +0.5 | +2.5 | 0.0058 | Milk | IgG1 |
|  |  |  |  |  |  |  | +0.8 | +0.0 | +1.6 | 0.0423 | Serum | IgG1 |
|  | Alum-Saponin-Oil | - | Boost | Alum-Saponin | -  - | Boost | +2.6 | +1.9 | +3.4 | <0.0001 | Serum | IgG1 |
|  |  |  |  |  |  |  | +2.5 | +1.4 | +3.5 | <0.0001 | Milk | IgG1 |
|  |  |  |  |  |  |  | +1.9 | +1.3 | +2.6 | <0.0001 | Serum | IgA |
|  |  |  |  |  |  |  | +1.6 | +0.7 | +2.5 | 0.0011 | Milk | IgA |
|  | Alum-Saponin-Oil | - | Boost | Alum-Oil | - | Boost | +1.8 | +1.0 | +2.6 | <0.0001 | Serum | IgG1 |
|  |  |  |  |  |  |  | +1.2 | +0.6 | +1.9 | 0.0006 | Serum | IgA |
|  |  |  |  |  |  |  | +1.2 | +0.3 | +2.2 | 0.0091 | Milk | IgA |
|  |  |  |  |  |  |  | +1.2 | +0.2 | +2.3 | 0.0266 | Milk | IgG1 |
|  | Alum-Oil | - | Boost | Alum-Saponin | - | Boost | +1.2 | +0.2 | +2.3 | 0.0175 | Milk | IgG1 |
|  |  |  |  |  |  |  | +0.9 | +0.1 | +1.7 | 0.0329 | Serum | IgG1 |
|  |  |  |  |  |  |  | +0.7 | +0.0 | +1.4 | 0.0405 | Serum | IgA |
| Adjuvant * Site | Alum-Saponin-Oil | Udder | - | Alum-Saponin | Udder | - | +2.6 | +1.4 | +3.8 | <0.0001 | Milk | IgG1 |
|  |  | Udder | - | Alum-Oil | Udder | - | +1.9 | +0.6 | +3.3 | 0.0069 | Milk | IgG1 |
|  |  | Neck | - | Alum-Saponin | Neck | - | +1.2 | +0.0 | +2.4 | 0.0441 | Milk | IgG1 |
|  | Alum-Oil | Neck | - | Alum-Saponin | Neck | - | +2.1 | +0.9 | +3.2 | 0.0009 | Milk | IgG1 |
|  |  | Udder | - | Alum-Oil | Neck | - | -2.1 | -3.4 | -0.9 | 0.0014 | Milk | IgG1 |
| Site * Time | - | Udder | Prime | - | Neck | Prime | +1.1 | +0.3 | +1.9 | 0.0068 | Milk | IgG2 |
|  |  |  |  |  |  |  | +0.9 | +0.4 | +1.5 | 0.0010 | Serum | IgG2 |
|  |  |  |  |  |  |  | +0.8 | +0.4 | +1.3 | <0.0001 | Serum | NT |
|  | - | Udder | Prime | - | Udder | Boost | -0.9 | -1.6 | -0.3 | 0.0068 | Milk | IgG1 |
|  |  |  |  |  |  |  | -0.9 | -1.2 | -0.6 | <0.0001 | Serum | IgA |
|  |  |  |  |  |  |  | -0.8 | -1.2 | -0.4 | 0.0003 | Milk | NT |
|  |  |  |  |  |  |  | -0.6 | -0.9 | -0.3 | 0.0004 | Serum | NT |
|  |  |  |  |  |  |  | -0.5 | -0.9 | -0.1 | 0.0132 | Serum | IgG1 |
|  |  |  |  |  |  |  | -0.5 | -0.9 | -0.0 | 0.0419 | Milk | IgG2 |
|  | - | Neck | Prime | - | Neck | Boost | -2.0 | -2.4 | -1.6 | <0.0001 | Milk | IgG2 |
|  |  |  |  |  |  |  | -1.9 | -2.6 | -1.3 | <0.0001 | Milk | IgG1 |
|  |  |  |  |  |  |  | -1.8 | -2.2 | -1.4 | <0.0001 | Milk | NT |
|  |  |  |  |  |  |  | -1.4 | -1.8 | -1.0 | <0.0001 | Serum | IgG1 |
|  |  |  |  |  |  |  | -1.4 | -1.7 | -1.2 | <0.0001 | Serum | IgA |
|  |  |  |  |  |  |  | -1.3 | -1.6 | -1.0 | 0.0008 | Serum | NT |
|  |  |  |  |  |  |  | -0.9 | -1.3 | -0.5 | <0.0001 | Serum | IgG2 |
|  | - | Udder | Boost | - | Neck | Boost | -1.2 | -2.0 | -0.4 | 0.0048 | Milk | IgG1 |
|  |  |  |  |  |  |  | -0.9 | -1.4 | -0.3 | 0.0028 | Milk | NT |
|  |  |  |  |  |  |  | -0.7 | -1.3 | -0.1 | 0.0364 | Serum | IgG1 |

NT = Neutralization Titer

^a^ # Compared to

^b^ Confidence Interval (95 %)
